# Risk of Death in Individuals Hospitalized for COVID-19 With and Without Psychiatric Disorders: An Observational Multicenter Study in France

**DOI:** 10.1016/j.bpsgos.2021.12.007

**Published:** 2022-01-04

**Authors:** Nicolas Hoertel, Marina Sánchez-Rico, Pedro de la Muela, Miriam Abellán, Carlos Blanco, Marion Leboyer, Céline Cougoule, Erich Gulbins, Johannes Kornhuber, Alexander Carpinteiro, Katrin Anne Becker, Raphaël Vernet, Nathanaël Beeker, Antoine Neuraz, Jesús M. Alvarado, Juan José Herrera-Morueco, Guillaume Airagnes, Cédric Lemogne, Frédéric Limosin, AP-HP/Université de Paris/Inserm COVID-19 Research Collaboration and the AP-HP COVID CDR Initiative, “Entrepôt de Données de Santé,” AP-HP Consortium, Pierre-Yves Ancel, Alain Bauchet, Nathanaël Beeker, Vincent Benoit, Mélodie Bernaux, Ali Bellamine, Romain Bey, Aurélie Bourmaud, Stéphane Breant, Anita Burgun, Fabrice Carrat, Charlotte Caucheteux, Julien Champ, Sylvie Cormont, Christel Daniel, Julien Dubiel, Catherine Ducloas, Loic Esteve, Marie Frank, Nicolas Garcelon, Alexandre Gramfort, Nicolas Griffon, Olivier Grisel, Martin Guilbaud, Claire Hassen-Khodja, François Hemery, Martin Hilka, Anne Sophie Jannot, Jerome Lambert, Richard Layese, Judith Leblanc, Léo Lebouter, Guillaume Lemaitre, Damien Leprovost, Ivan Lerner, Kankoe Levi Sallah, Aurélien Maire, Marie-France Mamzer, Patricia Martel, Arthur Mensch, Thomas Moreau, Antoine Neuraz, Nina Orlova, Nicolas Paris, Bastien Rance, Hélène Ravera, Antoine Rozes, Elisa Salamanca, Arnaud Sandrin, Patricia Serre, Xavier Tannier, Jean-Marc Treluyer, Damien Van Gysel, Gaël Varoquaux, Jill Jen Vie, Maxime Wack, Perceval Wajsburt, Demian Wassermann, Eric Zapletal

**Affiliations:** a Département Médico-Universitaire Psychiatrie et Addictologie, Service de Psychiatrie et Addictologie, Assistance Publique–Hôpitaux de Paris Centre, Hôpital Corentin-Celton, Issy-les-Moulineaux, France; b Institut National de la Santé et de la Recherche Médicale (INSERM) U1266, Paris, France; c Université de Paris, Paris, France; d Department of Medical Informatics, Biostatistics and Public Health Department, L'Assistance Publique–Hôpitaux de Paris Centre-Université de Paris, Hôpital Européen Georges Pompidou, Paris, France; e Unité de Recherche clinique, L'Assistance Publique–Hôpitaux de Paris, Hôpital Cochin, Paris, France; f INSERM UMR_S 1138, Cordeliers Research Center, Université de Paris, Paris, France; g INSERM U955, Neuro-Psychiatrie Translationnelle, Université Paris-Est, Paris, France; h Department of Medical Informatics, L'Assistance Publique–Hôpitaux de Paris, Necker-Enfants Malades Hospital, Paris, France; i Département Médico-Universitaire Psychiatrie et Addictologie, L'Assistance Publique–Hôpitaux de Paris, Hôpital Hôtel-Dieu, Université de Paris, Service de Psychiatrie de l’adulte, INSERM, Institut de Psychiatrie et Neurosciences de Paris, Paris, France; j Département Médico-Universitaire IMPACT, Département Médical Universitaire de Psychiatrie, Hôpitaux Universitaires Henri Mondor, Créteil, France; k Institut de Pharmacologie et de Biologie Structurale, Université de Toulouse, Toulouse, France; l Department of Psychobiology & Behavioural Sciences Methods, Faculty of Psychology, Universidad Complutense de Madrid, Campus de Somosaguas, Pozuelo de Alarcón, Spain; m Division of Epidemiology, Services and Prevention Research, National Institute on Drug Abuse, Bethesda, Maryland; n Department of Molecular Biology, University Medicine Essen, Essen, Germany; o Department of Hematology and Stem Cell Transplantation, University Hospital Essen, University of Duisburg-Essen, Essen, Germany; p Department of Psychiatry and Psychotherapy, University Hospital, Friedrich Alexander University of Erlangen Nuremberg, Erlangen, Germany

**Keywords:** Antidepressants, Comorbidity, COVID-19, Mental disorders, Mood disorders, Mortality, Obesity, Psychiatric disorders, Risk of death, SARS-CoV-2

## Abstract

**Background:**

Prior research suggests that psychiatric disorders could be linked to increased mortality among patients with COVID-19. However, whether all or specific psychiatric disorders are intrinsic risk factors of death in COVID-19 or whether these associations reflect the greater prevalence of medical risk factors in people with psychiatric disorders has yet to be evaluated.

**Methods:**

We performed an observational, multicenter, retrospective cohort study to examine the association between psychiatric disorders and mortality among patients hospitalized for laboratory-confirmed COVID-19 at 36 Greater Paris University hospitals.

**Results:**

Of 15,168 adult patients, 857 (5.7%) had an ICD-10 diagnosis of psychiatric disorder. Over a mean follow-up period of 14.6 days (SD = 17.9), 326 of 857 (38.0%) patients with a diagnosis of psychiatric disorder died compared with 1276 of 14,311 (8.9%) patients without such a diagnosis (odds ratio 6.27, 95% CI 5.40–7.28, *p* < .01). When adjusting for age, sex, hospital, current smoking status, and medications according to compassionate use or as part of a clinical trial, this association remained significant (adjusted odds ratio 3.27, 95% CI 2.78–3.85, *p* < .01). However, additional adjustments for obesity and number of medical conditions resulted in a nonsignificant association (adjusted odds ratio 1.02, 95% CI 0.84–1.23, *p* = .86). Exploratory analyses after the same adjustments suggested that a diagnosis of mood disorders was significantly associated with reduced mortality, which might be explained by the use of antidepressants.

**Conclusions:**

These findings suggest that the increased risk of COVID-19–related mortality in individuals with psychiatric disorders hospitalized for COVID-19 might be explained by the greater number of medical conditions and the higher prevalence of obesity in this population and not by the underlying psychiatric disease.

Prior studies (1, 2, 3, 4, 5, 6, 7, 8, 9, 10) suggest that psychiatric disorders, including schizophrenia spectrum disorders (1, 2, 3, 4,7), mood disorders (1, 2, 3,8), anxiety disorders (1), intellectual and developmental disabilities (9), substance-induced psychiatric disorders (1,2), and dementia (5), are associated with higher COVID-19–related mortality. However, premature mortality observed in patients with psychiatric disorders is usually attributable to comorbid medical illnesses (11,12), which themselves are associated with increased COVID-19–related mortality (13). Indeed, this association may be confounded by several demographic and medical risk factors including sex, age, ethnicity, obesity, and a history of certain medical comorbidities such as cardiovascular diseases, diabetes, kidney diseases, and asthma (1). Because these comorbidities are also associated with increased COVID-19–related mortality (13), it is important to determine whether psychiatric disorders are independent risk factors of death due to COVID-19 or whether this association is explained by the greater rates of medical risk factors for severe COVID-19 in this population.

This issue is key in the context of the worldwide infectious disease crisis (14, 15, 16), where limited resources, including the vaccine, are allocated based on the vulnerability to develop severe COVID-19. This knowledge is also important to advance in the identification of risk factors associated with poor COVID-19 outcomes, guide clinical decision making, target enhanced protective measures, and prevent increased health inequalities (4).

A recent meta-analysis (10) of 23 studies including 43,938 individuals with any psychiatric disorder and 1,425,793 control participants indicates that psychiatric disorders may be associated with an increased risk of death after SARS-CoV-2 infection (pooled unadjusted odds ratio [OR] 2.00, 95% CI 1.58–2.54), suggesting that psychiatric disorders per se may be intrinsic risk factors of death in COVID-19. However, only 9 of the 23 studies included in this meta-analysis adjusted for a limited number of comorbid medical conditions. Furthermore, few studies (4,8) examined the risk of mortality associated with psychiatric diagnosis in hospitalized patients with COVID-19. For example, Nemani *et al.* (4) found that a premorbid diagnosis of a schizophrenia spectrum disorder was significantly associated with increased mortality, and Castro *et al.* (8) reported a significant positive association between mood disorders and COVID-19 mortality. However, these prior studies included a restricted number of psychiatric disorders (i.e., schizophrenia spectrum disorders, anxiety disorders, and mood disorders), took into account a relatively limited number of medical risk factors (i.e., hypertension, diabetes, myocardial infarction, heart failure, chronic obstructive pulmonary disease, chronic kidney disease, cancer, and smoking status), and did not adjust for obesity. Therefore, the risk of mortality after SARS-CoV-2 infection in patients with psychiatric disorders while including a broader range of medical risk factors [i.e., obesity and other medical disorders (17)] has yet to be evaluated.

To address this knowledge gap, a multicenter, observational, retrospective cohort study was conducted in 36 Greater Paris University hospitals (18, 19, 20, 21). In this report, we examined the association between a diagnosis of psychiatric disorder and mortality in patients hospitalized for laboratory-confirmed COVID-19. Based on prior studies (1, 2, 3, 4, 5, 6, 7, 8, 9, 10,22), we hypothesized that mortality would be higher in all psychiatric diagnostic groups compared with patients without a diagnosis of a psychiatric disorder and that this association would be mainly explained by the greater prevalence of medical risk factors, including medical comorbidities and obesity, in patients with psychiatric disorders.

## Methods and Materials

### Setting and Cohort Assembly

We conducted a multicenter, observational, retrospective cohort study at 36 L’Assistance Publique–Hôpitaux de Paris (AP-HP) University hospitals from the onset of the epidemic in France, i.e., January 24, 2020, until May 1, 2020 (18, 19, 20, 21). We included all adults aged 18 years and older who had been admitted to one of these centers for laboratory-confirmed COVID-19. COVID-19 was ascertained by a positive reverse transcriptase polymerase chain reaction test from the analysis of nasopharyngeal or oropharyngeal swab specimens.

This observational study using routinely collected data received approval from the Institutional Review Board of the AP-HP Clinical Data Warehouse (decision CSE-20- 20_COVID19, IRB00011591, April 8, 2020). AP-HP Clinical Data Warehouse initiatives ensure patient information and consent regarding the different approved studies through a transparency portal in accordance with European Regulation on Data Protection and Authorization No. 1980120 from the National Commission for Information Technology and Civil Liberties.

### Data Sources

AP-HP Health Data Warehouse (Entrepôt de Données de Santé) contains all available clinical data on all inpatient visits for COVID-19 to 36 Greater Paris University hospitals. The data include patient demographic characteristics, vital signs, laboratory test and reverse transcriptase polymerase chain reaction test results, medication administration data, current medical diagnoses, and death certificates.

### Variables Assessed

Data for each patient were obtained at the time of the hospitalization through electronic health records (23). The assessments of the variables are detailed in the Supplement.

### Assessment of Psychiatric Disorders

Psychiatric disorder diagnoses were also obtained through electronic health records and recorded at the time of hospitalization for COVID-19, based on the ICD-10 diagnosis codes (F01–F99) as determined by the practitioners in charge of the patients. Patients with at least one ICD-10 diagnosis of mental, behavioral, or neurodevelopmental disorder (F01–F99) were considered as having a psychiatric disorder.

### Study Baseline and Outcome

The study baseline was defined as the date of hospital admission for COVID-19. The outcome was all-cause mortality from the study baseline until the end of the hospitalization for COVID-19 or until the time of data cutoff on May 1, 2020.

### Statistical Analysis

We calculated the frequencies of all baseline characteristics described above in patients with and without a diagnosis of psychiatric disorder and compared them using standardized mean differences.

To examine the crude, unadjusted association between a diagnosis of psychiatric disorder and all-cause mortality, we performed a logistic regression model. Patients with a diagnosis of psychiatric disorder were compared with a reference group without a diagnosis of psychiatric disorder. To reduce the effects of confounding, the primary analysis was a multivariable logistic regression that included sex, age, hospital, current smoking status, medications according to compassionate use or as part of a clinical trial, obesity, and the number of current medical comorbidities.

We performed several additional exploratory analyses. First, we reproduced these analyses for different psychiatric diagnostic categories (i.e., illness-induced psychiatric disorders [F01–F09]; substance-induced psychiatric disorders [F10–F19], which was subdivided into alcohol-induced [F10] and other substance-induced [F11–F19] psychiatric disorders (24); and primary psychiatric disorders [F20–F99]) and different psychiatric diagnoses (i.e., schizophrenia spectrum disorders [F20–F29]; mood disorders [F30–F39]; anxiety and other nonpsychotic disorders [F40–F48]; behavioral syndromes [F50–F59]; personality disorders [F60–F69]; intellectual disabilities [F70–F79]; developmental disorders [F80–F89]; behavioral and emotional disorders [F90–F98]; or unspecified psychiatric disorders [F99]). Patients with these diagnoses were successively compared with 1) a reference group without a diagnosis of psychiatric disorder and 2) a reference group with other psychiatric disorders.

If a significant positive association was found following adjustments for sociodemographic and medical risk factors, we planned to study it further following additional adjustments for clinical severity at baseline, i.e., at the time of hospital admission. Clinical severity was defined as having at least one of the following criteria at baseline (25,26): respiratory rate >24 breaths/min or <12 breaths/min, resting peripheral capillary oxygen saturation in ambient air <90%, temperature >40 °C, systolic blood pressure <100 mm Hg, and lactate levels >2 mmol/L.

If any significant protective association was found following the same adjustments, we planned to study it further in the subpopulations of patients with and without antidepressant use because these medications have been previously suggested to be potentially associated with reduced COVID-19–related mortality (27) in these (18) and other (28) data, in several preclinical studies (29, 30, 31), and in two clinical trials (32,33).

Second, we reproduced the main analyses after imputing missing data using multiple imputation. Third, we replicated the main analyses while categorizing the number of medical comorbidities into four classes (i.e., 0, 1–3, 4–6, and 6+) instead of three classes (i.e., 0–3, 4–6, and 6+) to distinguish the group of patients without any medical comorbidity. Fourth, we performed additional multivariable logistic regression models including interaction terms to examine whether the association between any psychiatric disorder and mortality significantly differed by age and sex. Fifth, we examined whether having at least two diagnoses of psychiatric disorders was associated with a significantly greater risk of death than having only one diagnosis of psychiatric disorder. Sixth, we examined, among patients with a diagnosis of any psychiatric disorder, whether the mortality risk differed between those with and without this diagnosis confirmed during a prior hospitalization in Greater Paris AP-HP hospitals in the past 2 years. Finally, we reproduced the main analyses while successively adjusting additionally for time to follow-up and individual medications prescribed as part of a clinical trial or according to compassionate use.

For all associations, we assessed the fit of the data, checked assumptions, and examined the potential influence of outliers. We followed the recommendations of the Strengthening the Reporting of Observational Studies in Epidemiology Initiative (34). Because our main hypothesis focused on the association between any psychiatric disorder and all-cause mortality, statistical significance was fixed a priori at a two-sided *p* value < .05. We planned to perform additional exploratory analyses as described above only if a significant association was found. All analyses were conducted in R software (version 3.6.3; R Project for Statistical Computing).

## Results

### Characteristics of the Cohort

**Figure 1:**
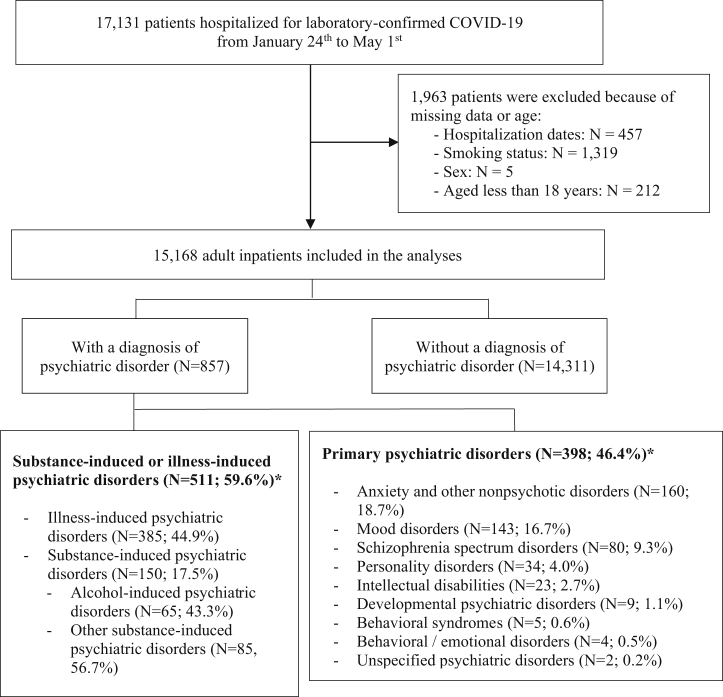
Study cohort. Individuals can have more than one diagnosis of psychiatric disorder. Percentages for each psychiatric diagnosis category refer to the total number of patients with any diagnosis of psychiatric disorder (*n* = 857).

Of the 17,131 patients hospitalized for laboratory-confirmed COVID-19, 1963 patients (11.5%) were excluded because of missing data or their young age (i.e., <18 years old). Of these 15,168 patients, 857 (5.7%) had a diagnosis of psychiatric disorder and 14,311 (94.3%) did not (Figure 1). Of these 857 patients, 511 (59.6%) patients had a substance- or illness-induced psychiatric disorder, 398 patients (46.4%) had a primary psychiatric disorder, and 52 patients (6.1%) had both types of disorders.

Reverse transcriptase polymerase chain reaction test results were obtained after a median delay of 1.2 days (SD = 12.7) from the hospital admission date. This median delay was not significantly different between patients with and without a diagnosis of a psychiatric disorder (0.9 days [SD = 12.4] vs. 1.2 days [SD = 12.7]; Brown-Mood median test, *Z* = 1.93; *p* = .053).

Over a mean follow-up of 14.6 days (SD = 17.9, median = 8 days), 1602 patients (10.6%) died at the time of data cutoff on May 1. Among patients who had a diagnosis of a psychiatric disorder, the mean follow-up was 12.7 days (SD = 12.4, median = 9 days), while it was 14.7 days (SD = 18.2, median = 7 days) in patients without a diagnosis of a psychiatric disorder (Welch two-sample *t* test, *t*_1012.6_ = 4.4, *p* < .001).

Each sociodemographic and medical risk factor was individually and significantly associated with mortality (Table S1). A multivariable logistic regression model showed that sex, age, hospital, obesity, and the number of conditions were significantly and independently associated with this outcome (Table S1).

The distributions of patient characteristics according to the psychiatric disorder diagnosis are shown in Table 1. Psychiatric disorders substantially differed according to all patient characteristics except for sex. The direction of the associations indicated an older age (proportions of patients older than 70 years: 67.8% vs. 27.5%), a greater prevalence of obesity (19.5% vs. 13.2%), and a greater number of medical conditions (proportions of patients with at least four medical comorbidities: 95.5% vs. 17.8%) in patients with a psychiatric disorder diagnosis than in those without this diagnosis.

**Table 1:** Characteristics of Patients Hospitalized for COVID-19 With and Without a Diagnosis of Psychiatric Disorder (*N* = 15,168)

Characteristics	With a Diagnosis of Psychiatric Disorder, *n* = 857, *n* (%)	Without a Diagnosis of Psychiatric Disorder, *n* = 14,311, *n* (%)	With a Diagnosis of Psychiatric Disorder vs. Without a Diagnosis of Psychiatric Disorder, Crude Analysis, SMD
Age, Years			1.020^a^
18–50	64 (7.47%)	5765 (40.3%)	
51–70	212 (24.7%)	4603 (32.2%)	
71–80	187 (21.8%)	1690 (11.8%)	
>80	394 (46.0%)	2253 (15.7%)	
Sex			0.077
Men	438 (51.1%)	6766 (47.3%)	
Women	419 (48.9%)	7545 (52.7%)	
Hospital			0.543^a^
AP-HP Centre—Paris University, Henri Mondor University Hospitals, and at home hospitalization	272 (31.7%)	6769 (47.3%)	
AP-HP Nord and Hôpitaux Universitaires Paris Seine-Saint-Denis, France	162 (18.9%)	3967 (27.7%)	
AP-HP Paris Saclay University	248 (28.9%)	1629 (11.4%)	
AP-HP Sorbonne University	175 (20.4%)	1946 (13.6%)	
Obesity^b^			0.169^a^
Yes	167 (19.5%)	1895 (13.2%)	
No	690 (80.5%)	12,416 (86.8%)	
Smoking^c^			0.303^a^
Yes	155 (18.1%)	1145 (8.0%)	
No	702 (81.9%)	13,166 (92.0%)	
Medications According to Compassionate Use or as Part of a Clinical Trial^d^			0.228^a^
Yes	170 (19.8%)	1660 (11.6%)	
No	687 (80.2%)	12,651 (88.4%)	
Number of Comorbid Medical Conditions^e^			2.625^a^
0–3	38 (4.5%)	11,759 (82.2%)	
4–6	307 (35.8%)	1794 (12.5%)	
≥6	512 (59.7%)	758 (5.3%)	

AP-HP, L'Assistance Publique–Hôpitaux de Paris; SMD, standardized mean difference.

a SMD > 0.1 indicates substantial difference.

b Defined as having a body mass index higher than 30 kg/m^2^ or an ICD-10 diagnosis code for obesity (E66.0, E66.1, E66.2, E66.8, E66.9).

c Current smoking status was self-reported.

d Any medication prescribed as part of a clinical trial or according to compassionate use (e.g., hydroxychloroquine, azithromycin, remdesivir, tocilizumab, sarilumab, or dexamethasone).

e Assessed using ICD-10 diagnosis codes for certain infectious and parasitic diseases (A00–B99); neoplasms (C00–D49); diseases of the blood and blood-forming organs and certain disorders involving the immune mechanism (D50–D89); diseases of the nervous system (G00–G99); diseases of the circulatory system (I00–I99); diseases of the respiratory system (J00–J99); diseases of the digestive system (K00–K95); diseases of the skin and subcutaneous tissue (L00–L99); diseases of the musculoskeletal system and connective tissue (M00–M99); diseases of the genitourinary system (N00–N99); endocrine, nutritional, and metabolic diseases (E00–E89); diseases of the eye and adnexa (H00–H59); and diseases of the ear and mastoid process (H60–H95).

### Association Between Psychiatric Disorders and Mortality

**Figure 2:**
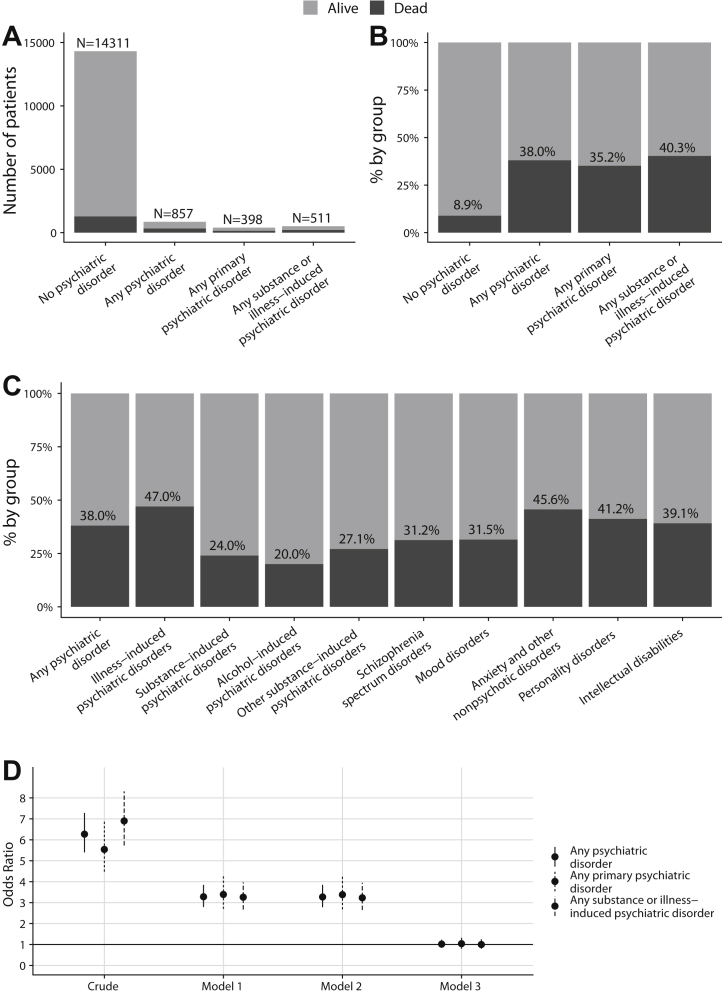
Number of patients hospitalized for COVID-19 with and without a diagnosis of psychiatric disorder **(A)**, mortality rates by psychiatric diagnosis category **(B)** and by psychiatric diagnosis **(C)**, and associations between a diagnosis of psychiatric disorder and mortality **(D)**. Only psychiatric diagnoses with more than 20 patients are displayed in **(C)**. In **(D)**, model 1 was adjusted for age and sex; model 2 was adjusted for age, sex, hospital, current smoking, and medications according to compassionate use or as part of a clinical trial; and model 3 was adjusted for age, sex, hospital, current smoking, medications according to compassionate use or as part of a clinical trial, obesity, and number of medical conditions.

Death occurred in 326 of 857 (38.0%) patients with a diagnosis of psychiatric disorder and in 1276 of 14,311 (8.9%) patients without this diagnosis. Rates of mortality by psychiatric diagnosis category ranged from 20.0% in patients with an alcohol-induced psychiatric disorder to 47.0% in patients with an illness-induced psychiatric disorder (Figure 2 and Table 2).

**Table 2:** Associations of Each Diagnostic Category and Each Diagnosis of Psychiatric Disorder With Mortality Among Patients Hospitalized for COVID-19 (*N* = 15,168)

Diagnoses	Number of Events/Number of Patients (%)	Crude Logistic Regression Analysis, OR (95% CI); *p* Value	Multivariable Logistic Regression Analysis,^a^ AOR (95% CI); *p* Value	Multivariable Logistic Regression Analysis,^b^ AOR (95% CI); *p* Value	Multivariable Logistic Regression Analysis,^c^ AOR (95% CI); *p* Value
No Psychiatric Disorder	1276/14,311 (8.9%)	Ref.	Ref.	Ref.	Ref.
Any Psychiatric Disorder^d^	326/857 (38.0%)	6.27 (5.40–7.28); <.001^e^	3.28 (2.79–3.85); <.001^e^	3.27 (2.78–3.85); <.001^e^	1.02 (0.84–1.23); .855
Substance-Induced or Illness-Induced Psychiatric Disorders	206/511 (40.3%)	6.90 (5.73–8.31); <.001^e^	3.26 (2.67–3.97); <.001^e^	3.23 (2.65–3.95); <.001^e^	1.00 (0.80–1.25); .984
Illness-induced psychiatric disorders^f^	181/385 (47.0%)	9.06 (7.36–11.16); <.001^e^	3.69 (2.96–4.60); <.001^e^	3.72 (2.98–4.65); <.001^e^	1.15 (0.90–1.47); .276
Substance-induced psychiatric disorders^g^	36/150 (24.0%)	3.23 (2.21–4.71); <.001^e^	2.26 (1.50–3.41); <.001^e^	2.15 (1.43–3.24); <.001^e^	0.64 (0.42–0.99); .046^e^
Alcohol-induced psychiatric disorders	13/65 (20.0%)	2.55 (1.39–4.70); .003^e^	1.71 (0.89–3.29); .106	1.62 (0.85–3.10); .144	0.52 (0.26–1.03); .060
Substance-induced psychiatric disorders	23/85 (27.1%)	3.79 (2.34–6.14); <.001^e^	2.74 (1.63–4.63); <.001^e^	2.60 (1.55–4.38); <.001^e^	0.73 (0.42–1.26); .262
Primary Psychiatric Disorders	140/398 (35.2%)	5.54 (4.48–6.86); <.001^e^	3.39 (2.70–4.27); <.001^e^	3.38 (2.68–4.26); <.001^e^	1.04 (0.80–1.35); .765
Schizophrenia spectrum disorders^h^	25/80 (31.2%)	4.64 (2.88–7.48); <.001^e^	3.61 (2.16–6.05); <.001^e^	3.64 (2.17–6.11); <.001^e^	1.27 (0.74–2.19); .382
Mood disorders^i^	45/143 (31.5%)	4.69 (3.28–6.71); <.001^e^	2.31 (1.59–3.37); <.001^e^	2.31 (1.59–3.38); <.001^e^	0.66 (0.44–0.99); .045^e^
Anxiety and other nonpsychotic disorders^j^	73/160 (45.6%)	8.57 (6.25–11.76); <.001^e^	5.11 (3.62–7.22); <.001^e^	5.07 (3.59–7.17); <.001^e^	1.43 (0.99–2.07); .058
Behavioral syndromes^k^	1/5 (20.0%)	NA	NA	NA	NA
Personality disorders^l^	14/34 (41.2%)	7.15 (3.60–14.19); <.001^e^	3.09 (1.50–6.37); .002^e^	3.37 (1.63–6.98); .001^e^	0.84 (0.40–1.80); .663
Intellectual disabilities^m^	9/23 (39.1%)	6.57 (2.84–15.20); <.001^e^	8.45 (3.41–20.9); <.001^e^	8.17 (3.3–20.24); <.001^e^	1.70 (0.59–4.88); .324
Developmental psychiatric disorders^n^	0/9 (0.0%)	NA	NA	NA	NA
Behavioral and emotional disorders^o^	1/4 (25.0%)	NA	NA	NA	NA
Unspecified psychiatric disorders^p^	1/2 (50.0%)	NA	NA	NA	NA

AOR, adjusted odds ratio; NA, not applicable; OR, odds ratio.

a Adjusted for age and sex.

b Adjusted for age, sex, hospital, smoking, and medications according to compassionate use or as part of a clinical trial.

c Adjusted for age, sex, hospital, smoking, medications according to compassionate use or as part of a clinical trial, obesity, and number of medical conditions.

d Assessed using ICD-10 diagnosis codes for mental, behavioral and neurodevelopmental disorders (F01–F99).

e Two-sided *p* value is significant (*p* < .05).

f Assessed using ICD-10 diagnosis codes for psychiatric disorders due to known physiological conditions (F01–F09).

g Assessed using ICD-10 diagnosis codes for mental and behavioral disorders due to psychoactive substance use (F10–F19).

h Assessed using ICD-10 diagnosis codes for schizophrenia, schizotypal, delusional, and other nonmood psychotic disorders (F20–F29).

i Assessed using ICD-10 diagnosis codes for mood (affective) disorders (F30–F39).

j Assessed using ICD-10 diagnosis codes for anxiety, dissociative, stress-related, somatoform, and other nonpsychotic psychiatric disorders (F40–F49).

k Assessed using ICD-10 diagnosis codes for behavioral syndromes associated with physiological disturbances and physical factors (F50–F59).

l Assessed using ICD-10 diagnosis codes for disorders of adult personality and behavior (F60–F69).

m Assessed using ICD-10 diagnosis codes for intellectual disabilities (F70–F79).

n Assessed using ICD-10 diagnosis codes for pervasive and specific developmental psychiatric disorders (F80–F89).

o Assessed using ICD-10 diagnosis codes for behavioral and emotional disorders with onset usually occurring in childhood and adolescence (F90–F98).

p Assessed using ICD-10 diagnosis codes for unspecified psychiatric disorder (F99).

The crude unadjusted analysis (OR 6.27, 95% CI 5.40–7.28, *p* < .001), the multivariable regression analysis adjusted for age and sex (adjusted OR [AOR] 3.28, 95% CI 2.79–3.85; *df* = 5, *p* < .001), and the multivariable regression analysis adjusted for age, sex, hospital, current smoking status, and medications according to compassionate use or as part of a clinical trial (AOR 3.27, 95% CI 2.78–3.85, *df* = 10, *p* < .001) showed significant association between a diagnosis of a psychiatric disorder and increased mortality. However, this association was not significant after additional adjustments for obesity and the number of medical conditions (AOR 1.02, 95% CI 0.84–1.23, *df* = 13, *p* = .855) (Table 2).

Exploratory analyses showed that all psychiatric diagnosis categories and all individual diagnoses of psychiatric disorders were significantly associated with increased mortality following adjustments for age, sex, hospital, current smoking status, and medications according to compassionate use or as part of a clinical trial. However, following additional adjustments for obesity and the number of medical conditions, no psychiatric diagnosis category or individual diagnosis of psychiatric disorder was significantly associated with increased mortality (Table 2). Rather, following these adjustments, mood disorders and substance-induced psychiatric disorders were significantly associated with reduced mortality (AOR 0.66, 95% CI 0.44–0.99, *p* = .045 and AOR 0.64, 95% CI 0.42–0.99, *p* = .046, respectively) (Table 2). Additional analyses further adjusting for clinical severity at baseline indicated that the association between a diagnosis of mood disorders and decreased mortality was significant in patients with mood disorders receiving an antidepressant during the visit, whereas this association was not significant in those not taking an antidepressant (Table S2). After stratifying for antidepressant use, substance-induced psychiatric disorders were not significantly associated with mortality.

When examining these associations in the population of patients with a diagnosis of a psychiatric disorder, we found that mortality rates were significantly higher in patients with diagnoses of anxiety disorders and intellectual disabilities than in those diagnosed with other psychiatric disorders (Table 3). These two associations remained significant after additional adjustment for baseline clinical severity (Table S3). Conversely, a diagnosis of mood disorders was significantly associated with lower mortality compared with a diagnosis of other psychiatric disorders (Table 3). This association was not statistically significant when stratifying for antidepressant use, possibly because of less statistical power (Table S2).

**Table 3:** Associations of Each Diagnostic Category and Each Diagnosis of Psychiatric Disorder With Mortality Among Patients With a Diagnosis of Psychiatric Disorder Hospitalized for COVID-19 (*n* = 857)

Diagnoses	Number of Events/Number of Patients (%)	Crude Logistic Regression Analysis, OR (95% CI); *p* Value	Multivariable Logistic Regression Analysis,^a^ AOR (95% CI); *p* Value	Multivariable Logistic Regression Analysis,^b^ AOR (95% CI); *p* Value	Multivariable Logistic Regression Analysis,^c^ AOR (95% CI); *p* Value
Other Psychiatric Disorders	120/346 (34.7%)	Ref.	Ref.	Ref.	Ref.
Substance-induced or illness-induced psychiatric disorder	206/511 (40.3%)	1.27 (0.96–1.69); .096	1.01 (0.74–1.37); .965	1.03 (0.75–1.41); .858	1.03 (0.75–1.42); .862
Other Psychiatric Disorders	145/472 (30.7%)	Ref.	Ref.	Ref.	Ref.
Illness-induced psychiatric disorder^d^	181/385 (47.0%)	2.00 (1.51–2.65); <.001^e^	1.23 (0.90–1.68); .187	1.28 (0.93–1.75); .130	1.30 (0.95–1.78); .104
Other Psychiatric Disorders	290/707 (41.0%)	Ref.	Ref.	Ref.	Ref.
Substance-induced psychiatric disorder^f^	36/150 (24.0%)	0.45 (0.30–0.68); <.001^e^	0.72 (0.45–1.13); .153	0.66 (0.40–1.07); .088	0.62 (0.38–1.02); .058
Other Psychiatric Disorders	313/792 (39.5%)	Ref.	Ref.	Ref.	Ref.
Alcohol-induced psychiatric disorders (F10)	13/65 (20.0%)	0.38 (0.2–0.71); .003^e^	0.57 (0.29–1.11); .099	0.54 (0.27–1.07); .078	0.55 (0.27–1.1); .091
Other Psychiatric Disorders	303/772 (39.2%)	Ref.	Ref.	Ref.	Ref.
Substance-induced psychiatric disorders	23/85 (27.1%)	0.57 (0.35–0.95); .030^e^	0.92 (0.53–1.61); .769	0.87 (0.48–1.55); .631	0.79 (0.44–1.43); .439
Other Psychiatric Disorders	186/459 (40.5%)	Ref.	Ref.	Ref.	Ref.
Primary psychiatric disorder	140/398 (35.2%)	0.80 (0.60–1.05); .108	1.08 (0.80–1.46); .628	1.09 (0.79–1.49); .605	1.10 (0.8–1.51); .559
Other Psychiatric Disorders	301/777 (38.7%)	Ref.	Ref.	Ref.	Ref.
Schizophrenia spectrum disorder^g^	25/80 (31.2%)	0.72 (0.44–1.18); .191	1.23 (0.72–2.12); .452	1.22 (0.70–2.1); .486	1.32 (0.75–2.31); .331
Other Psychiatric Disorders	281/714 (39.4%)	Ref.	Ref.	Ref.	Ref.
Mood disorders^h^	45/143 (31.5%)	0.71 (0.48–1.04); .077	0.64 (0.42–0.96); .032^e^	0.63 (0.42–0.96); .030^e^	0.62 (0.41–0.95); .027^e^
Other Psychiatric Disorders	253/697 (36.3%)	Ref.	Ref.	Ref.	Ref.
Anxiety and other nonpsychotic disorders^i^	73/160 (45.6%)	1.47 (1.04–2.08); .029^e^	1.67 (1.14–2.45); .008^e^	1.63 (1.1–2.41); .014^e^	1.63 (1.10–2.41); .014^e^
Other Psychiatric Disorders	325/852 (38.1%)	Ref.	Ref.	Ref.	Ref.
Behavioral syndromes^j^	1/5 (20.0%)	NA	NA	NA	NA
Other Psychiatric Disorders	312/823 (37.9%)	Ref.	Ref.	Ref.	Ref.
Personality disorders^k^	14/34 (41.2%)	1.15 (0.57–2.30); .701	0.87 (0.41–1.81); .704	0.82 (0.39–1.74); .606	0.79 (0.37–1.68); .537
Other Psychiatric Disorders	317/834 (38.0%)	Ref.	Ref.	Ref.	Ref.
Intellectual disabilities^l^	9/23 (39.1%)	1.05 (0.45–2.45); .913	3.03 (1.20–7.62); .019^e^	3.19 (1.24–8.22); .016^e^	3.36 (1.30–8.71); .012^e^
Other Psychiatric Disorders	326/848 (38.4%)	Ref.	Ref.	Ref.	Ref.
Developmental disorders^m^	0/9 (0%)	NA	NA	NA	NA
Other Psychiatric Disorders	325/853 (38.1%)	Ref.	Ref.	Ref.	Ref.
Behavioral and emotional disorders^n^	1/4 (25.0%)	NA	NA	NA	NA
Other Psychiatric Disorders	325/855 (38.0%)	Ref.	Ref.	Ref.	Ref.
Unspecified psychiatric disorder^o^	1/2 (50.0%)	NA	NA	NA	NA

AOR, adjusted odds ratio; NA, not applicable; OR, odds ratio.

a Adjusted for age and sex.

b Adjusted for age, sex, hospital, smoking, and medications according to compassionate use or as part of a clinical trial.

c Adjusted for age, sex, hospital, smoking, medications according to compassionate use or as part of a clinical trial, obesity, and number of medical conditions.

d Assessed using ICD-10 diagnosis codes for psychiatric disorders due to known physiological conditions (F01–F09).

e Two-sided *p* value is significant (*p* < .05).

f Assessed using ICD-10 diagnosis codes for mental and behavioral disorders due to psychoactive substance use (F10–F19).

g Assessed using ICD-10 diagnosis codes for schizophrenia, schizotypal, delusional, and other nonmood psychotic disorders (F20–F29).

h Assessed using ICD-10 diagnosis codes for mood (affective) disorders (F30–F39).

i Assessed using ICD-10 diagnosis codes for anxiety, dissociative, stress-related, somatoform, and other nonpsychotic psychiatric disorders (F40–F49).

j Assessed using ICD-10 diagnosis codes for behavioral syndromes associated with physiological disturbances and physical factors (F50–F59).

k Assessed using ICD-10 diagnosis codes for disorders of adult personality and behavior (F60–F69).

l Assessed using ICD-10 diagnosis codes for intellectual disabilities (F70–F79).

m Assessed using ICD-10 diagnosis codes for pervasive and specific developmental psychiatric disorders (F80–F89).

n Assessed using ICD-10 diagnosis codes for behavioral and emotional disorders with onset usually occurring in childhood and adolescence (F90–F98).

o Assessed using ICD-10 diagnosis codes for unspecified psychiatric disorder (F99).

Results of additional analyses are detailed in Tables S4–S12.

## Discussion

In this multicenter, retrospective, observational study involving 15,168 patients hospitalized for laboratory-confirmed COVID-19, we found that individuals with a diagnosis of a psychiatric disorder had a sixfold increased risk of mortality than those without this diagnosis. All individual psychiatric diagnoses were significantly associated with increased mortality when adjusting for age, sex, hospital, current smoking status, and medications according to compassionate use or as part of a clinical trial. However, after additionally adjusting for obesity and the number of medical comorbidities, no diagnosis of psychiatric disorder was significantly associated with mortality as compared with individuals without psychiatric disorders. A notable exception was that a diagnosis of mood disorders was significantly associated with reduced mortality, which might be explained by antidepressant use. Our analyses suggest that increased mortality in patients diagnosed with a psychiatric disorder compared with those without this diagnosis was mainly explained by the higher rates of medical risk factors, including a greater number of medical conditions and the higher prevalence of obesity, in this population.

Of the 15,168 patients hospitalized for COVID-19, we found that 857 (5.7%) had a diagnosis of a psychiatric disorder, of which 398 (46.4%) had a primary psychiatric disorder, 511 (59.6%) had a substance-induced or illness-induced psychiatric disorder, and 52 (6.1%) had both types of disorders. Although no direct comparison can be performed in this study, the rate of any current psychiatric disorder in our sample was about twofold lower than that observed in the European or French general population, estimated at 11.5% (35) and between 9.6% (36) and 14.5% (37), respectively. In particular, the prevalence of any current mood disorder was 0.9% in our sample, contrasting with the rates observed in the European or French general population, estimated at 4.2% (35) and between 4.3% (36) and 6.7% (37), respectively. Because people with psychiatric disorders might be at a higher risk of contracting COVID-19 (1), possibly because of lower adherence to barrier measures and socioeconomic and lifestyle factors, we can hypothesize that a substantial proportion of the people with psychiatric disorders might have a reduced risk of severe COVID-19 requiring hospitalization, possibly owing to certain psychotropic treatments such as antidepressants, as previously suggested (18,27,29,32,33), whereas those with a high number of comorbid medical disorders and/or obesity could be at a higher risk of developing severe COVID-19. However, an alternative explanation for these results includes a potential underreporting of the diagnoses of psychiatric disorders in patients hospitalized for COVID-19, for whom the clinical priority was the treatment of the infection, especially in the context of overwhelmed hospital units during peak incidence. Future longitudinal studies involving outpatients and inpatients with COVID-19 with and without psychiatric disorders are required to examine this issue.

In line with prior evidence (1, 2, 3, 4, 5, 6, 7, 8, 9, 10), our results show that among patients hospitalized for COVID-19, those with a diagnosis of a psychiatric disorder had an increased risk of mortality than those without this diagnosis. Because this observation was common to all psychiatric disorders, our results support the need for considering these patients as a vulnerable population that requires specific care and for targeting them for interventions and distribution of resources, including screening and priority for COVID-19 vaccines (38).

Our results indicate that this increased risk of mortality associated with psychiatric disorders could be explained by the higher rates of medical risk factors, including a greater number of medical conditions and higher prevalence of obesity, which importantly increase the risk of severe COVID-19 (13,17,39). These findings suggest that treatment of general medical conditions may decrease mortality due to COVID-19 in this population. Treatment of psychiatric disorders may also help improve the general medical care of these patients and therefore may also decrease mortality. Therefore, prevention and treatment of medical risk factors of severe COVID-19 through collaborative primary and mental health care should be intensified during the COVID-19 pandemic to reduce morbidity and mortality associated with COVID-19 in this population and help prevent deepened health inequalities (3,4).

No category of psychiatric disorder was significantly associated with increased mortality compared with patients without psychiatric disorders. This finding supports the idea that it is not psychiatric disorders per se that increase this risk but rather specific determinants of health, including social, economic, and environmental factors, which may influence both mental and physical health and mortality risk in COVID-19. For example, obesity and medical diseases, a number of which are due to alcohol or tobacco consumption, may constitute shared risk factors of both psychiatric disorders and COVID-19–related mortality.

One notable exception was that patients diagnosed with mood disorders, specifically those taking an antidepressant during the visit, showed a reduced risk of death than patients without psychiatric disorders, which was not explained by differences in medical risk factors or clinical severity at baseline. The hypothesis previously advanced, that of potential antiviral and anti-inflammatory effects of several psychotropic medications, especially those of certain antidepressants such as fluoxetine or fluvoxamine, which are taken by a substantial proportion of people with mood disorders, could be a promising avenue to explore further (27,40). For example, preclinical (29, 30, 31,41,42), observational (18,28,43), and clinical (32,33) trial findings support that fluvoxamine and fluoxetine may be associated with better outcomes in patients with COVID-19. A randomized placebo-controlled clinical trial (32) showed that a 2-week prescription of fluvoxamine was associated with a reduced risk of clinical deterioration in outpatients with COVID-19, showing that decrease in anxiety may not explain these results because antidepressants are usually not effective for anxiety in the very short term (44), and anxiety is unlikely to reduce oxygen saturation (32,33).

Several mechanisms have been proposed to explain the potential beneficial effect of certain antidepressants against COVID-19 (27,45). First, these treatments belong to the group of functional inhibitors of acid sphingomyelinase called FIASMA (46,47), which in vitro and in vivo inhibit acid sphingomyelinase, an enzyme that catalyzes the hydrolysis of sphingomyelin into ceramide and phosphorylcholine (29,46,48). According to preclinical evidence, SARS-CoV-2 activates the acid sphingomyelinase/ceramide system, resulting in the formation of ceramide-enriched membrane domains that may facilitate viral entry and infection by clustering ACE2, the cellular receptor of SARS-CoV-2 (29,48). Several observational studies also show that hospitalized patients with COVID-19 taking FIASMA medications may have a reduced risk of death (43,47,49,50). Other potential mechanisms include the anti-inflammatory properties of antidepressants (51,52) with sigma-1 receptor agonist effect (32,45,53) and/or decreasing acid sphingomyelinase activity (29,48), which may have an important value in regulating inflammation by inhibiting cytokine production in COVID-19, reduction in platelet aggregation, decreased mast cell degranulation, interference with endolysosomal viral trafficking, and increased melatonin levels (27,45). Finally, alternative explanations to this result include statistical artifacts owing to multiple testing or differential access and timing to general physical care (54,55).

Among patients with a diagnosis of psychiatric disorder, mortality rates may be significantly higher in those with diagnoses of anxiety disorders or intellectual disabilities. These results are in line with prior findings (56, 57, 58). We may hypothesize that individuals with intellectual disabilities are more likely to have genetic conditions linked to higher risk for other medical conditions. Individuals with intellectual disabilities or anxiety disorders may also be less likely to adequately communicate their symptoms and distress to care providers (57, 58, 59, 60). Patient complaints may also be wrongly attributed to these conditions or considered as exaggerated in these patients, despite their elevated risk of death as suggested by our findings, which may lead to less thorough medical evaluation and worse prognosis. Finally, it is also possible that anxiety could be more frequent among patients with more severe COVID-19 illness or that anxiety disorder may be a resulting sequela of severe COVID-19 in some patients (61). Future studies are required to help understand the determinants of these potential associations.

Our study has several limitations. First, an inherent bias in observational studies is unmeasured confounding. We tried to minimize the effects of confounding and performed the analyses while adjusting for numerous potential confounders. Second, there were missing data for some baseline characteristic variables (i.e., 11.5%), which might be explained by the overwhelming of all hospital units during COVID-19 peak incidence, and different results might have been observed during a lower COVID-19 incidence period. However, sensitivity analyses using imputed missing data reached similar results. Third, because of the cross-sectional design, correlation does not imply causation (62). Fourth, inflation of type I error might have occurred in secondary exploratory analyses due to multiple testing. Fifth, there is a potential underreporting of psychiatric disorders and medical comorbidities in our sample in the context of overwhelmed hospital units during peak incidence. However, this bias is unlikely to explain the associations observed between psychiatric disorder diagnoses and mortality. Sixth, the precise date of the diagnosis of psychiatric disorders during the visit (e.g., at hospital admission or at the end of the visit) was not available. Seventh, diagnoses of psychiatric disorders were based on ICD-10 diagnosis codes made by practitioners in charge of the patients during hospitalization for COVID-19 and were not ascertained by psychiatrists. Finally, despite the multicenter design, our results relied on a cohort study of hospitalized patients with COVID-19, whose study period was from January 24, 2020, to May 1, 2020, and whose study follow-up period was from hospital admission until the end of the hospitalization for COVID-19 or until the time of data cutoff on May 1, 2020. In addition, we lacked information on certain important sociodemographic characteristics such as ethnicity, country of birth, or migrant status. Our findings may thus not be generalizable to outpatients and other countries, longer follow-up periods, and more recent periods during which prevention and care have substantially progressed, limiting public health recommendations to the general population.

Findings from this multicenter retrospective observational study suggested an increased risk of mortality in individuals diagnosed with a psychiatric disorder than in those without this diagnosis, which could be explained by higher rates of medical risk factors, including a greater number of medical conditions and higher prevalence of obesity, in this population. Future studies including data on outpatients and inpatients with and without psychiatric disorders and COVID-19 and taking into account main medical risk factors of severe COVID-19, i.e., age, medical comorbidities, and obesity, will be important to determine whether the risk of hospitalization and mortality due to SARS-CoV-2 infection is similar or different across psychiatric diagnoses and psychotropic medications prescribed.

## Supplementary Material

Supplementary Material

Key Resources Table
