# Supplementary material for: Risk of Death in Individuals Hospitalized for COVID-19 With and Without Psychiatric Disorders: An Observational Multicenter Study in France
Source: Biol Psychiatry Glob Open Sci. 2022 Jan 4;3(1):56–67. doi: 10.1016/j.bpsgos.2021.12.007 (PMC8730644; doi:10.1016/j.bpsgos.2021.12.007)
Supplement: Supplementary Material [file mmc1.pdf]

Risk of Death in Individuals Hospitalized for COVID-19 With and Without Psychiatric Disorders: An Observational Multicenter Study in France

SUPPLEMENTARY INFORMATION

Supplementary text

Variables assessed

We obtained the following data for each patient at the time of the hospitalization through electronic health records (1): sex; age, which was categorized into 4 groups based on the OpenSAFELY study results (2) (i.e., 18-50, 51-70, 71-80, 81+); hospital, which was categorized into 4 classes following the administrative clustering of AP-HP hospitals in Paris and its suburbs based on their geographical location (i.e., AP-HP Centre – Paris University, Henri Mondor University Hospitals and at home hospitalization; AP-HP Nord and Hôpitaux Universitaires Paris Seine-Saint-Denis; AP-HP Paris Saclay University; and AP-HP Sorbonne University); obesity, which was defined as having a body mass index higher than 30 kg/m^2 or an International Statistical Classification of Diseases and Related Health Problems (ICD-10) diagnosis code for obesity (E66.0, E66.1, E66.2, E66.8, E66.9); self-reported current smoking status; any medication prescribed according to compassionate use or as part of a clinical trial (e.g. hydroxychloroquine, azithromycin, remdesivir, tocilizumab, sarilumab, or dexamethasone); and number of medical comorbidities, based on ICD-10 diagnosis codes recorded at the time of hospitalization for COVID-19. The variable 'number of medical comorbidities' was categorized into 3 classes (i.e., 0-3, 4-6, 6+) based on a tertile split in the population with a diagnosis of psychiatric disorder.

Results of additional analyses

When categorizing the number of medical comorbidities into 4 classes (i.e., 0, 1-3, 4-6, 6+) instead of 3 classes (i.e., 0-3, 4-6, 6+) to distinguish the group of patients without any medical comorbidity, results remained unchanged, except for anxiety disorders and intellectual disabilities, which were not significantly associated with mortality (**Table S4**). There were no significant differences across age groups or by sex in the association between any psychiatric disorder and mortality (**Table S5**). Having at least two diagnoses of psychiatric disorders was not significantly associated with a different risk of death than having a single diagnosis of psychiatric disorder (**Table S6**). Among patients with a diagnosis of any psychiatric disorder, those with such diagnosis already mentioned during a prior hospitalization in Greater Paris AP-HP hospitals in the last two years were significantly at higher risk of death than those who had a diagnosis of psychiatric disorder only during the current stay. However, following adjustments, the difference in mortality was not statistically significant between these patients and those without a diagnosis of psychiatric disorders (**Table S7**). Reproducing the main analyses after imputing missing data did not modify the results (**Tables S8 and S9**). Finally, results from the main analyses were similar when adjusting additionally for time to follow-up or individual medications prescribed as part of a clinical trial or according to compassionate use (**Tables S10 to S12**).

Table S1. Association of socio-demographic and medical risk factors with mortality among patients hospitalized for COVID-19 (N=15,168).

	Full sample (N=15,168)	Death (N=1,602)	Alive (N=13,566)	Crude logistic regression analyses	Multivariable logistic regression analysis
	N (%)	N (%)	N (%)	OR (95%CI; p-value)	AOR (95%CI; p-value)
Age					
18 to 50 years	5829 (38.4%)	57 (3.6%)	5772 (42.5%)	Ref.	Ref.
51 to 70 years	4815 (31.7%)	371 (23.2%)	4444 (32.8%)	8.45 (6.38 – 11.20; <0.001*)	4.88 (3.66 - 6.51; <0.001*)
71 to 80 years	1877 (12.4%)	401 (25.0%)	1476 (10.9%)	27.51 (20.72 – 36.52; <0.001*)	13.73 (10.26 - 18.37; <0.001*)
More than 80 years	2647 (17.5%)	773 (48.3%)	1874 (13.8%)	41.77 (31.76 - 54.94; <0.001*)	28.72 (21.7 - 38.02; <0.001*)
Sex					
Men	7204 (47.5%)	996 (62.2%)	6208 (45.8%)	1.95 (1.75 – 2.17; <0.001*)	1.76 (1.55 – 2.00; <0.001*)
Women	7964 (52.5%)	606 (37.8%)	7358 (54.2%)	Ref.	Ref.
Hospital					
AP-HP Centre - Paris University, Henri Mondor University Hospitals and at home hospitalization	7041 (46.4%)	518 (32.3%)	6523 (48.1%)	Ref.	Ref.
AP-HP Nord and Hôpitaux Universitaires Paris Seine-Saint-Denis	4129 (27.2%)	526 (32.8%)	3603 (26.6%)	1.84 (1.62 - 2.09; <0.001*)	1.53 (1.32 - 1.77; <0.001*)
AP-HP Paris Saclay University	1877 (12.4%)	292 (18.2%)	1585 (11.7%)	2.32 (1.99 - 2.70; <0.001*)	0.97 (0.81 - 1.16; 0.749)
AP-HP Sorbonne University	2121 (14.0%)	266 (16.6%)	1855 (13.7%)	1.81 (1.54 - 2.11; <0.001*)	0.94 (0.79 - 1.13; 0.535)
Obesity [a]					
Yes	2062 (13.6%)	347 (21.7%)	1715 (12.6%)	1.91 (1.68 – 2.17; <0.001*)	1.42 (1.22 - 1.65; <0.001*)
No	13106 (86.4%)	1255 (78.3%)	11851 (87.4%)	Ref.	Ref.
Smoking [b]					
Yes	1300 (8.6%)	238 (14.9%)	1062 (7.8%)	2.05 (1.77 – 2.39; <0.001*)	1.11 (0.93 - 1.32; 0.250)
No	13868 (91.4%)	1364 (85.1%)	12504 (92.2%)	Ref.	Ref.
Medications according to compassionate use or as part of a clinical trial [c]					
Yes	1830 (12.1%)	319 (19.9%)	1511 (11.1%)	1.98 (1.74 – 2.27; <0.001*)	1.00 (0.85 - 1.17; 0.964)
No	13338 (87.9%)	1283 (80.1%)	12055 (88.9%)	Ref.	Ref.
Number of medical conditions [d]					
0 to 3	11797 (77.8%)	607 (37.9%)	11190 (82.5%)	Ref.	Ref.
4 to 6	2101 (13.9%)	468 (29.2%)	1633 (12.0%)	5.28 (4.63 - 6.02; <0.001*)	3.88 (3.35 - 4.49; <0.001*)
6 or more	1270 (8.37%)	527 (32.9%)	743 (5.48%)	13.08 (11.39 - 15.02; <0.001*)	7.77 (6.65 - 9.08; <0.001*)

[a] Defined as having a body-mass index higher than 30 kg/m^2 or an International Statistical Classification of Diseases and Related Health Problems (ICD-10) diagnosis code for obesity (E66.0, E66.1, E66.2, E66.8, E66.9).
[b] Current Smoking status was self-reported.

[c] Any medication prescribed as part of a clinical trial or according to compassionate use (e.g., hydroxychloroquine, azithromycin, remdesivir, tocilizumab, sarilumab or dexamethasone).
[d] Assessed using ICD-10 diagnosis codes for certain infectious and parasitic diseases (A00-B99); neoplasms (C00-D49); diseases of the blood and blood-forming organs and certain disorders involving the immune mechanism (D50-D89); diseases of the nervous system (G00-G99); diseases of the circulatory system (I00-I99); diseases of the respiratory system (J00-J99); diseases of the digestive system (K00-K95); diseases of the skin and subcutaneous tissue (L00-L99); diseases of the musculoskeletal system and connective tissue (M00-M99); diseases of the genitourinary system (N00-N99); endocrine, nutritional and metabolic diseases (E00-E89); diseases of the eye and adnexa (H00-H59) and diseases of the ear and mastoid process (H60-H95)
* Two-sided p-value is significant ($p<0.05$).
Abbreviations: OR, odds ratio; AOR, adjusted odds ratio; CI, confidence interval.

Table S2. Associations of mood disorders and substance-induced psychiatric disorders with mortality in patients hospitalized for COVID-19, by antidepressant use during the visit.

	Number of events / Number of patients	Crude logistic regression analysis	Multivariable logistic regression analysis [a]	Multivariable logistic regression analysis [b]	Multivariable logistic regression analysis [c]
	N / N (%)	OR (95% CI; p-value)	AOR (95% CI; p-value)	AOR (95% CI; p-value)	AOR (95% CI; p-value)
**With antidepressant during the hospitalization** [N=921 (6.1%)]					
No psychiatric disorder	151 / 668 (22.6)	Ref.	Ref.	Ref.	Ref.
Substance-induced psychiatric disorder [d]	8 / 25 (32.0)	1.61 (0.68 - 3.81; 0.277)	1.83 (0.75 - 4.44; 0.182)	0.70 (0.27 - 1.87; 0.481)	0.78 (0.29 - 2.10; 0.624)
Mood disorders [e]	23 / 90 (25.6)	1.18 (0.71 - 1.95; 0.532)	1.13 (0.68 - 1.9; 0.631)	0.47 (0.26 - 0.86; 0.014*)	0.52 (0.28 - 0.94; 0.032*)
**Without antidepressant during the hospitalization** [N=14,247 (93.9%)]					
No psychiatric disorder	1125 / 13643 (8.2)	Ref.	Ref.	Ref.	Ref.
Substance-induced psychiatric disorder [d]	28 / 125 (22.4)	3.21 (2.10 - 4.91; <0.001*)	2.14 (1.35 - 3.39; 0.001*)	0.61 (0.37 - 0.99; 0.046*)	0.68 (0.42 - 1.11; 0.122)
Mood disorders [e]	22 / 53 (41.5)	7.9 (4.56 - 13.68; <0.001*)	4.14 (2.33 - 7.36; <0.001*)	1.15 (0.62 - 2.11; 0.663)	1.43 (0.77 - 2.64; 0.259)
**With a diagnosis of psychiatric disorder and an antidepressant during the hospitalization** [N=253 (29.5%)]					
Other psychiatric disorders	56 / 163 (34.4)	Ref.	Ref.	Ref.	Ref.
Mood disorders [e]	23 / 90 (25.6)	0.66 (0.37 - 1.16; 0.149)	0.60 (0.33 - 1.08; 0.091)	0.60 (0.32 - 1.12; 0.111)	0.63 (0.33 - 1.19; 0.151)
**With a diagnosis of psychiatric disorder and no antidepressant during the hospitalization** [N=604 (70.5%)]					
Other psychiatric disorders	225 / 551 (40.8)	Ref.	Ref.	Ref.	Ref.
Mood disorders [e]	22 / 53 (41.5)	1.03 (0.58 - 1.82; 0.924)	1.05 (0.56 - 1.97; 0.873)	0.93 (0.49 - 1.76; 0.819)	1.08 (0.55 - 2.13; 0.818)

[a] Adjusted for age and sex.
[b] Adjusted for age, sex, hospital, smoking, medications according to compassionate use or as part of a clinical trial, obesity, and number of medical conditions.
[c] Adjusted for age, sex, hospital, smoking, medications according to compassionate use or as part of a clinical trial, obesity, number of medical conditions, and clinical severity at baseline.
[d] Assessed using ICD-10 diagnosis codes for mental and behavioral disorders due to psychoactive substance use (F10-F19).

[e] Assessed using ICD-10 diagnosis codes for mood [affective] disorders (F30-F39).
* Two-sided p-value is significant ($p<0.05$).
Abbreviations: OR, odds ratio; AOR, adjusted odds ratio; CI, confidence interval.

Table S3. Associations of anxiety and other nonpsychotic disorders and intellectual disabilities with mortality in patients with psychiatric disorders hospitalized for COVID-19, following adjustment for clinical severity at baseline.

	**Number of events / Number of patients**	**Multivariable logistic regression analysis [a]**	**Multivariable logistic regression analysis [b]**
	N / N (%)	AOR (95% CI; p-value)	AOR (95% CI; p-value)
Other psychiatric disorders [N=695]	253 / 697 (36.3%)	Ref.	Ref.
Anxiety and other nonpsychotic disorders [c] [N=160]	73 / 160 (45.6%)	1.66 (1.12 - 2.46; 0.011*)	1.55 (1.03 - 2.33; 0.037*)
Other psychiatric disorders [N=832]	315 / 832 (37.9%)	Ref.	Ref.
Intellectual disabilities [d] [N=23]	9 / 23 (39.1%)	3.57 (1.36 - 9.37; 0.010*)	3.24 (1.17 - 8.97; 0.024*)

[a] Adjusted for age, sex, hospital, smoking, medications according to compassionate use or as part of a clinical trial, obesity, number of medical conditions, and clinical severity at baseline.
[b] Adjusted for age, sex, hospital, smoking, medications according to compassionate use or as part of a clinical trial, obesity, number of medical conditions, clinical severity at baseline, any antidepressant, any benzodiazepine or Z-drug, any antipsychotic, and any mood stabilizer medication.
[c] Assessed using ICD-10 diagnosis codes for anxiety, dissociative, stress-related, somatoform and other nonpsychotic psychiatric disorders (F40-F49).
[d] Assessed using ICD-10 diagnosis codes for intellectual disabilities (F70-F79).
* Two-sided p-value is significant (p<0.05).
Abbreviations: AOR, adjusted odds ratio; CI, confidence interval.

Table S4. Associations of each diagnostic category and each diagnosis of psychiatric disorder with mortality among patients hospitalized for COVID-19 (N=15,168), while including a category for 0 comorbidities for the number of relevant medical conditions.

	**Number of events / Number of patients**	**Multivariable logistic regression analysis** [a]
	N / N (%)	AOR (95% CI; p-value)
No psychiatric disorder	1276 / 14311 (8.9%)	Ref.
Any psychiatric disorder [b]	326 / 857 (38.0%)	0.98 (0.81 - 1.19; 0.870)
Substance-induced or illness-induced psychiatric disorders	206 / 511 (40.3%)	0.96 (0.77 - 1.21; 0.755)
Illness-induced psychiatric disorders [c]	181 / 385 (47.0%)	1.10 (0.86 - 1.41; 0.437)
Substance-induced psychiatric disorders [d]	36 / 150 (24.0%)	0.63 (0.41 - 0.98; 0.040*)
Alcohol induced psychiatric disorders [e]	13 / 65 (20.0%)	0.51 (0.26 - 1.01; 0.053)
Substance-induced psychiatric disorders[f]	23 / 85 (27.1%)	0.73 (0.42 - 1.25; 0.252)
Primary psychiatric disorders	140 / 398 (35.2%)	1.01 (0.78 - 1.31; 0.927)
Schizophrenia spectrum disorders [g]	25 / 80 (31.2%)	1.24 (0.72 - 2.13; 0.436)
Mood disorders [h]	45 / 143 (31.5%)	0.65 (0.43 - 0.97; 0.034*)
Anxiety and other nonpsychotic disorders [i]	73 / 160 (45.6%)	1.38 (0.95 - 1.99; 0.089)
Behavioral syndromes [j]	1 / 5 (20.0%)	NA
Personality disorders [k]	14 / 34 (41.2%)	0.82 (0.39 - 1.76; 0.616)
Intellectual disabilities [l]	9 / 23 (39.1%)	1.57 (0.50 - 4.91; 0.440)
Developmental psychiatric disorders [m]	0 / 9 (0.0%)	NA
Behavioral and emotional disorders [n]	1 / 4 (25.0%)	NA
Unspecified psychiatric disorders [o]	1 / 2 (50.0%)	NA

[a] Adjusted for age, sex, hospital, smoking, medications according to compassionate use or as part of a clinical trial, as well as obesity and number of medical conditions.
[b] Assessed using ICD-10 diagnosis codes for mental, behavioral and neurodevelopmental disorders (F01-F99).
[c] Assessed using ICD-10 diagnosis codes for psychiatric disorders due to known physiological conditions (F01-F09).

[d] Assessed using ICD-10 diagnosis codes for mental and behavioral disorders due to psychoactive substance use (F10-F19).
[e] Assessed using ICD-10 diagnosis codes for alcohol related disorders (F10)
[f] Assessed using ICD-10 diagnosis codes for (F11-F19)
[g] Assessed using ICD-10 diagnosis codes for schizophrenia, schizotypal, delusional, and other non-mood psychotic disorders (F20-F29).
[h] Assessed using ICD-10 diagnosis codes for mood [affective] disorders (F30-F39).
[i] Assessed using ICD-10 diagnosis codes for anxiety, dissociative, stress-related, somatoform and other nonpsychotic psychiatric disorders (F40-F49).
[j] Assessed using ICD-10 diagnosis codes for behavioral syndromes associated with physiological disturbances and physical factors (F50-F59).
[k] Assessed using ICD-10 diagnosis codes for disorders of adult personality and behavior (F60-F69).
[l] Assessed using ICD-10 diagnosis codes for intellectual disabilities (F70-F79).
[m] Assessed using ICD-10 diagnosis codes for pervasive and specific developmental psychiatric disorders (F80-F89).
[n] Assessed using ICD-10 diagnosis codes for behavioral and emotional disorders with onset usually occurring in childhood and adolescence (F90-F98).
[o] Assessed using ICD-10 diagnosis codes for unspecified psychiatric disorder (F99).
* Two-sided p-value is significant (p<0.05).
Abbreviations: OR, odds ratio; AOR, adjusted odds ratio; CI, confidence interval, NA, not applicable.

Table S5. Effect of sex and age on the association between any psychiatric disorder and mortality among patients hospitalized for COVID-19 (N=15,168).

	Multivariable[a] logistic models including successively all characteristics and the corresponding interaction term (characteristic*any psychiatric disorder [b])
	HR (95% CI; p-value)
Characteristics	
Age (years)	
18-50	Ref.
51-70	0.48 (0.15 - 1.48; 0.201)
71-80	0.47 (0.15 - 1.44; 0.187)
81+	0.58 (0.19 - 1.72; 0.325)
Sex	
Women	Ref.
Men	1.01 (0.73 - 1.41; 0.941)

[a] Adjusted for age, sex, hospital, smoking, medications according to compassionate use or as part of a clinical trial, as well as obesity and number of medical conditions.
[b] Assessed using ICD-10 diagnosis codes for mental, behavioural and neurodevelopmental disorders (F01-F99).
* Two-sided p-value is significant (p<0.05).
Abbreviations: OR, odds ratio; AOR, adjusted odds ratio; CI, confidence interval, NA, not applicable.

Table S6. Comparing the risk of death between patients hospitalized for COVID-19 with at least two psychiatric diagnoses versus those with a single psychiatric diagnosis.

	Number of events / Number of patients	Crude logistic regression analysis	Multivariable logistic regression analysis [a]	Multivariable logistic regression analysis [b]	Multivariable logistic regression analysis [c]
	N / N (%)	OR (95% CI; p-value)	AOR (95% CI; p-value)	AOR (95% CI; p-value)	AOR (95% CI; p-value)
No psychiatric disorder	1276 / 14311 (8.9%)	Ref.	Ref.	Ref.	Ref.
One psychiatric disorder [d]	253 / 700 (36.1%)	5.78 (4.90 - 6.82; <0.001*)	3.07 (2.57 - 3.66; <0.001*)	3.04 (2.55 - 3.64; <0.001*)	0.93 (0.76 - 1.14; 0.506)
Two or more psychiatric disorders [d]	73 / 157 (46.5%)	8.88 (6.45 - 12.21; <0.001*)	4.31 (3.07 - 6.04; <0.001*)	4.46 (3.17 - 6.28; <0.001*)	1.24 (0.86 - 1.79; 0.241)
One psychiatric disorder [d]	253 / 700 (36.1%)	Ref.	Ref.	Ref.	Ref.
Two or more psychiatric disorders [d]	73 / 157 (46.5%)	1.54 (1.08 - 2.18; 0.016*)	1.40 (0.96 - 2.04; 0.077)	1.35 (0.92 - 1.98; 0.124)	1.33 (0.90 - 1.96; 0.150)

[a] Adjusted for age and sex.
[b] Adjusted for age, sex, hospital, smoking, medications according to compassionate use or as part of a clinical trial.
[c] Adjusted for age, sex, hospital, smoking, medications according to compassionate use or as part of a clinical trial, as well as obesity and number of medical conditions.
[d] Assessed using ICD-10 diagnosis codes for mental, behavioural and neurodevelopmental disorders (F01-F99).
* Two-sided p-value is significant (p<0.05).
Abbreviations: OR, odds ratio; AOR, adjusted odds ratio; CI, confidence interval, NA, not applicable.

Table S7. Comparing the risk of death between patients with a diagnosis of psychiatric disorder confirmed during a prior hospitalization in AP-HP Greater Paris University hospitals in the last two years versus those with a diagnosis of psychiatric disorder only during the hospitalization for COVID-19.

	Number of events / Number of patients	Crude logistic regression analysis	Multivariable logistic regression analysis [a]	Multivariable logistic regression analysis [b]	Multivariable logistic regression analysis [c]
	N / N (%)	OR (95% CI; p-value)	AOR (95% CI; p-value)	AOR (95% CI; p-value)	AOR (95% CI; p-value)
No psychiatric disorder	1276 / 14311 (8.9%)	Ref.	Ref.	Ref.	Ref.
Any psychiatric disorder without previous diagnosis [d]	195 / 585 (33.3%)	5.11 (4.26 - 6.12; <0.001*)	2.89 (2.38 - 3.50; <0.001*)	2.85 (2.35 - 3.47; <0.001*)	0.87 (0.69 - 1.08; 0.198)
At least one diagnosis of psychiatric disorder 2 years prior to this hospitalization [d]	131 / 272 (48.2%)	9.49 (7.43 - 12.12; <0.001*)	4.19 (3.23 - 5.44; <0.001*)	4.31 (3.31 - 5.60; <0.001*)	1.23 (0.92 - 1.64; 0.155)
Any psychiatric disorder without previous diagnosis [d]	195 / 585 (33.3%)	Ref.	Ref.	Ref.	Ref.
At least one diagnosis of psychiatric disorder 2 years prior to this hospitalization [d]	131 / 272 (48.2%)	1.86 (1.39 - 2.49; <0.001*)	1.42 (1.04 - 1.95; 0.029*)	1.43 (1.03 - 1.97; 0.031*)	1.44 (1.04 – 2.00; 0.027*)

[a] Adjusted for age and sex.
[b] Adjusted for age, sex, hospital, smoking, medications according to compassionate use or as part of a clinical trial.
[c] Adjusted for age, sex, hospital, smoking, medications according to compassionate use or as part of a clinical trial, as well as obesity and number of medical conditions.
[d] Assessed using ICD-10 diagnosis codes for mental, behavioural and neurodevelopmental disorders (F01-F99).
* Two-sided p-value is significant (p<0.05).
Abbreviations: OR, odds ratio; AOR, adjusted odds ratio; CI, confidence interval, NA, not applicable.

Table S8. Associations of each diagnostic category and each diagnosis of psychiatric disorder with mortality among patients hospitalized for COVID-19 after data imputation (N=16,919).

	Number of events / Number of patients	Crude logistic regression analysis	Multivariable logistic regression analysis [a]	Multivariable logistic regression analysis [b]	Multivariable logistic regression analysis [c]
	N / N (%)	OR (95% CI; p-value)	AOR (95% CI; p-value)	AOR (95% CI; p-value)	AOR (95% CI; p-value)
No psychiatric disorder	1447 / 16037 (9.0%)	Ref.	Ref.	Ref.	Ref.
Any psychiatric disorder [d]	334 / 882 (37.9%)	6.15 (5.31 - 7.11; <0.001*)	3.10 (2.65 - 3.63; <0.001*)	3.06 (2.61 - 3.59; <0.001*)	0.96 (0.79 - 1.15; 0.650)
Substance-induced or illness-induced psychiatric disorders	210 / 525 (40%)	6.72 (5.6 - 8.07; <0.001*)	3.06 (2.52 - 3.72; <0.001*)	3 (2.47 - 3.66; <0.001*)	0.93 (0.74 - 1.16; 0.522)
Illness-induced psychiatric disorders [e]	181 / 389 (46.5%)	8.77 (7.14 - 10.79; <0.001*)	3.42 (2.75 - 4.26; <0.001*)	3.43 (2.75 - 4.27; <0.001*)	1.06 (0.83 - 1.36; 0.619)
Substance-induced psychiatric disorders [f]	40 / 161 (24.8%)	3.33 (2.32 - 4.78; <0.001*)	2.24 (1.51 - 3.3; <0.001*)	2.1 (1.42 - 3.1; <0.001*)	0.62 (0.41 - 0.94; 0.024*)
Alcohol induced psychiatric disorders (F10)	16 / 72 (22.2%)	2.88 (1.65 - 5.03; <0.001*)	1.91 (1.05 - 3.48; 0.034*)	1.81 (1 - 3.28; 0.052)	0.57 (0.31 - 1.06; 0.078)
Substance-induced psychiatric disorders (Alcohol not included)	29 / 113 (25.7%)	3.48 (2.27 - 5.33; <0.001*)	2.31 (1.46 - 3.66; <0.001*)	2.16 (1.37 - 3.41; 0.001*)	0.6 (0.37 - 0.97; 0.037*)
Primary psychiatric disorders	144 / 411 (35%)	5.44 (4.41 - 6.71; <0.001*)	3.21 (2.56 - 4.03; <0.001*)	3.15 (2.51 - 3.97; <0.001*)	0.99 (0.77 - 1.27; 0.933)
Schizophrenia spectrum disorders [g]	28 / 86 (32.6%)	4.87 (3.09 - 7.67; <0.001*)	3.66 (2.24 - 5.99; <0.001*)	3.67 (2.24 - 6.02; <0.001*)	1.3 (0.78 - 2.18; 0.316)
Mood disorders [h]	46 / 144 (31.9%)	4.73 (3.32 - 6.75; <0.001*)	2.25 (1.55 - 3.26; <0.001*)	2.21 (1.51 - 3.21; <0.001*)	0.65 (0.44 - 0.97; 0.033*)
Anxiety and other nonpsychotic disorders [i]	73 / 165 (44.2%)	8 (5.86 - 10.93; <0.001*)	4.58 (3.26 - 6.43; <0.001*)	4.49 (3.2 - 6.31; <0.001*)	1.29 (0.9 - 1.85; 0.173)

Behavioral syndromes [j]	1 / 6 (16.7%)	NA	NA	NA	NA
Personality disorders [k]	14 / 34 (41.2%)	7.06 (3.56 - 14; <0.001*)	2.91 (1.41 - 6; 0.004*)	3.15 (1.52 - 6.53; 0.002*)	0.81 (0.38 - 1.73; 0.591)
Intellectual disabilities [l]	9 / 23 (39.1%)	6.48 (2.8 - 15; <0.001*)	7.86 (3.18 - 19.44; <0.001*)	7.61 (3.07 - 18.87; <0.001*)	2.95 (1.16 - 7.51; 0.024*)
Developmental psychiatric disorders [m]	0 / 9 (0%)	NA	NA	NA	NA
Behavioral and emotional disorders [n]	1 / 4 (25%)	NA	NA	NA	NA
Unspecified psychiatric disorders [o]	1 / 2 (50%)	NA	NA	NA	NA

[a] Adjusted for age and sex.
[b] Adjusted for age, sex, hospital, smoking, medications according to compassionate use or as part of a clinical trial.
[c] Adjusted for age, sex, hospital, smoking, medications according to compassionate use or as part of a clinical trial, as well as obesity and number of medical conditions.
[d] Assessed using ICD-10 diagnosis codes for mental, behavioural and neurodevelopmental disorders (F01-F99).
[e] Assessed using ICD-10 diagnosis codes for psychiatric disorders due to known physiological conditions (F01-F09).
[f] Assessed using ICD-10 diagnosis codes for mental and behavioral disorders due to psychoactive substance use (F10-F19).
[g] Assessed using ICD-10 diagnosis codes for schizophrenia, schizotypal, delusional, and other non-mood psychotic disorders (F20-F29).
[h] Assessed using ICD-10 diagnosis codes for mood [affective] disorders (F30-F39).
[i] Assessed using ICD-10 diagnosis codes for anxiety, dissociative, stress-related, somatoform and other nonpsychotic psychiatric disorders (F40-F49).
[j] Assessed using ICD-10 diagnosis codes for behavioral syndromes associated with physiological disturbances and physical factors (F50-F59).
[k] Assessed using ICD-10 diagnosis codes for disorders of adult personality and behavior (F60-F69).
[l] Assessed using ICD-10 diagnosis codes for intellectual disabilities (F70-F79).
[m] Assessed using ICD-10 diagnosis codes for pervasive and specific developmental psychiatric disorders (F80-F89).
[n] Assessed using ICD-10 diagnosis codes for behavioral and emotional disorders with onset usually occurring in childhood and adolescence (F90-F98).
[o] Assessed using ICD-10 diagnosis codes for unspecified psychiatric disorder (F99).
* Two-sided p-value is significant (p<0.05).
Abbreviations: OR, odds ratio; AOR, adjusted odds ratio; CI, confidence interval, NA, not applicable.

Table S9. Associations of each diagnostic category and each diagnosis of psychiatric disorder with mortality among patients with a diagnosis of psychiatric disorder hospitalized for COVID-19 after data imputation (N=882).

	Number of events / Number of patients	Crude logistic regression analysis	Multivariable logistic regression analysis [a]	Multivariable logistic regression analysis [b]	Multivariable logistic regression analysis [c]
	N / N (%)	OR (95% CI; p-value)	AOR (95% CI; p-value)	AOR (95% CI; p-value)	AOR (95% CI; p-value)
Other psychiatric disorders	124 / 357 (34.7%)	Ref.	Ref.	Ref.	Ref.
Substance-induced or illness-induced psychiatric disorder	210 / 525 (40%)	1.25 (0.95 - 1.66; 0.114)	1 (0.74 - 1.36; 0.98)	1.03 (0.75 - 1.4; 0.872)	1.02 (0.75 - 1.4; 0.897)
Other psychiatric disorders	153 / 493 (31%)	Ref.	Ref.	Ref.	Ref.
Illness-induced psychiatric disorder [d]	181 / 389 (46.5%)	1.93 (1.47 - 2.55; <0.001*)	1.18 (0.87 - 1.6; 0.286)	1.21 (0.89 - 1.65; 0.229)	1.23 (0.9 - 1.68; 0.193)
Other psychiatric disorders	294 / 721 (40.8%)	Ref.	Ref.	Ref.	Ref.
Substance-induced psychiatric disorder [e]	40 / 161 (24.8%)	0.48 (0.33 - 0.71; <0.001*)	0.78 (0.5 - 1.2; 0.258)	0.73 (0.46 - 1.16; 0.186)	0.69 (0.43 - 1.11; 0.125)
Other psychiatric disorders	318 / 810 (39.3%)	Ref.	Ref.	Ref.	Ref.
Alcohol induced psychiatric disorders (F10)	16 / 72 (22.2%)	0.44 (0.25 - 0.78; 0.005*)	0.69 (0.37 - 1.29; 0.246)	0.66 (0.35 - 1.25; 0.204)	0.67 (0.35 - 1.27; 0.216)
Other psychiatric disorders	305 / 769 (39.7%)	Ref.	Ref.	Ref.	Ref.
Substance-induced psychiatric disorders (Alcohol not included)	29 / 113 (25.7%)	0.53 (0.34 - 0.82; 0.005*)	0.83 (0.5 - 1.36; 0.454)	0.77 (0.45 - 1.3; 0.328)	0.7 (0.41 - 1.2; 0.194)
Other psychiatric disorders	190 / 471 (40.3%)	Ref.	Ref.	Ref.	Ref.
Primary psychiatric disorder	144 / 411 (35%)	0.8 (0.61 - 1.05; 0.106)	1.07 (0.79 - 1.44; 0.674)	1.07 (0.79 - 1.46; 0.65)	1.08 (0.79 - 1.48; 0.608)
Other psychiatric disorders	306 / 796 (38.4%)	Ref.	Ref.	Ref.	Ref.
Schizophrenia spectrum disorder [f]	28 / 86 (32.6%)	0.77 (0.48 - 1.24; 0.286)	1.32 (0.78 - 2.21; 0.302)	1.29 (0.76 - 2.19; 0.339)	1.39 (0.82 - 2.38; 0.224)
Other psychiatric disorders	288 / 738 (39.0%)	Ref.	Ref.	Ref.	Ref.
Mood disorders [g]	46 / 144 (31.9%)	0.73 (0.50 - 1.07; 0.110)	0.65 (0.44 - 0.98; 0.039*)	0.65 (0.43 - 0.97; 0.037*)	0.64 (0.42 - 0.97; 0.034*)

Other psychiatric disorders	261 / 717 (36.4%)	Ref.	Ref.	Ref.	Ref.
Anxiety and other nonpsychotic disorders [h]	73 / 165 (44.2%)	1.39 (0.98 - 1.95; 0.062)	1.54 (1.06 - 2.25; 0.023*)	1.55 (1.06 - 2.26; 0.024*)	1.51 (1.03 - 2.21; 0.034*)
Other psychiatric disorders	333 / 876 (38%)	Ref.	Ref.	Ref.	Ref.
Behavioral syndromes [i]	1 / 6 (16.7%)	NA	NA	NA	
Other psychiatric disorders	320 / 848 (37.7%)	Ref.	Ref.	Ref.	Ref.
Personality disorders [j]	14 / 34 (41.2%)	1.15 (0.58 - 2.32; 0.685)	0.86 (0.41 - 1.79; 0.677)	0.8 (0.38 - 1.69; 0.553)	0.77 (0.36 - 1.63; 0.49)
Other psychiatric disorders	325 / 859 (37.8%)	Ref.	Ref.	Ref.	Ref.
Intellectual disabilities [k]	9 / 23 (39.1%)	1.06 (0.45 - 2.47; 0.899)	2.88 (1.15 - 7.19; 0.024*)	2.97 (1.16 - 7.59; 0.023*)	3.12 (1.21 - 8.01; 0.018*)
Other psychiatric disorders	334 / 873 (38.3%)	Ref.	Ref.	Ref.	Ref.
Developmental disorders [l]	0 / 9 (0%)	NA	NA	NA	NA
Other psychiatric disorders	333 / 878 (37.9%)	Ref.	Ref.	Ref.	Ref.
Behavioral and emotional disorders [m]	1 / 4 (25%)	NA	NA	NA	NA
Other psychiatric disorders	333 / 880 (37.8%)	Ref.	Ref.	Ref.	Ref.
Unspecified psychiatric disorder [n]	1 / 2 (50%)	NA	NA	NA	NA

[a] Adjusted for age and sex.
[b] Adjusted for age, sex, hospital, smoking, medications according to compassionate use or as part of a clinical trial.
[c] Adjusted for age, sex, hospital, smoking, medications according to compassionate use or as part of a clinical trial, as well as obesity and number of medical conditions.
[d] Assessed using ICD-10 diagnosis codes for psychiatric disorders due to known physiological conditions (F01-F09).
[e] Assessed using ICD-10 diagnosis codes for mental and behavioral disorders due to psychoactive substance use (F10-F19).
[f] Assessed using ICD-10 diagnosis codes for schizophrenia, schizotypal, delusional, and other non-mood psychotic disorders (F20-F29).
[g] Assessed using ICD-10 diagnosis codes for mood [affective] disorders (F30-F39).
[h] Assessed using ICD-10 diagnosis codes for anxiety, dissociative, stress-related, somatoform and other nonpsychotic psychiatric disorders (F40-F49).
[i] Assessed using ICD-10 diagnosis codes for behavioral syndromes associated with physiological disturbances and physical factors (F50-F59).
[j] Assessed using ICD-10 diagnosis codes for disorders of adult personality and behavior (F60-F69).
[k] Assessed using ICD-10 diagnosis codes for intellectual disabilities (F70-F79).
[l] Assessed using ICD-10 diagnosis codes for pervasive and specific developmental psychiatric disorders (F80-F89).
[m] Assessed using ICD-10 diagnosis codes for behavioral and emotional disorders with onset usually occurring in childhood and adolescence (F90-F98).
[n] Assessed using ICD-10 diagnosis codes for unspecified psychiatric disorder (F99).
* Two-sided p-value is significant ($p<0.05$).
Abbreviations: OR, odds ratio; AOR, adjusted odds ratio; CI, confidence interval, NA, not applicable.

Table S10. Associations of psychiatric disorders with mortality in patients hospitalized for COVID-19, adjusting for time to follow-up.

	**Number of events / Number of patients**	**Multivariable logistic regression analysis [a]**
	N / N (%)	AOR (95% CI; p-value)
No psychiatric disorder	1276 / 14311 (8.9%)	Ref.
Any psychiatric disorder	326 / 857 (38.0%)	0.99 (0.81 - 1.19; 0.890)
Substance-induced psychiatric disorders [b]	36 / 150 (24.0%)	0.64 (0.41 - 0.98; 0.042*)
Mood disorders [c]	45 / 143 (31.5%)	0.65 (0.43 - 0.97; 0.034*)
Other psychiatric disorders	281 / 714 (39.4%)	Ref.
Mood disorders [c]	45 / 143 (31.5%)	0.63 (0.42 - 0.97; 0.034*)
Other psychiatric disorders	253 / 697 (36.3%)	Ref.
Anxiety and other nonpsychotic disorders [d]	73 / 160 (45.6%)	1.64 (1.11 - 2.44; 0.014*)
Other psychiatric disorders	317 / 834 (38.0%)	Ref.
Intellectual disabilities [e]	9 / 23 (39.1%)	3.33 (1.27 - 8.77; 0.015*)

[a] Adjusted for age, sex, hospital, smoking, medications according to compassionate use or as part of a clinical trial, obesity, number of medical conditions, and time to follow-up.
[b] Assessed using ICD-10 diagnosis codes for mental and behavioral disorders due to psychoactive substance use (F10-F19).
[c] Assessed using ICD-10 diagnosis codes for mood [affective] disorders (F30-F39).
[d] Assessed using ICD-10 diagnosis codes for anxiety, dissociative, stress-related, somatoform and other nonpsychotic psychiatric disorders (F40-F49).
[e] Assessed using ICD-10 diagnosis codes for intellectual disabilities (F70-F79).
* Two-sided p-value is significant (p<0.05).
Abbreviations: AOR, adjusted odds ratio; CI, confidence interval.

Table S11. Medications prescribed as part of a clinical trial or according to compassionate use in patients hospitalized for COVID-19 with and without a diagnosis of psychiatric disorder (N=15,168).

	With a diagnosis of psychiatric disorder (N=857)	Without a diagnosis of psychiatric disorder (N=14311)	With a diagnosis of psychiatric disorder vs. Without a diagnosis of psychiatric disorder
			Crude analysis
	N (%)	N (%)	SMD
Hydroxychloroquine	84 (9.8%)	1046 (7.3%)	0.089
Azithromycin	96 (11.2%)	779 (5.4%)	**0.210**
Remdesivir	1 (0.1%)	27 (0.2%)	0.018
Tocilizumab	23 (2.7%)	153 (1.1%)	**0.119**
Sarilumab	4 (0.5%)	54 (0.4%)	0.014
Dexamethasone	8 (0.9%)	137 (1.0%)	0.002

Table S12. Associations of psychiatric disorders with mortality in patients hospitalized for COVID-19, adjusting for individual medications prescribed as part of a clinical trial or according to compassionate use.

	**Number of events / Number of patients**	**Multivariable logistic regression analysis [a]**
	N / N (%)	AOR (95% CI; p-value)
No psychiatric disorders	1276 / 14311 (8.9%)	Ref.
Any psychiatric disorder	326 / 857 (38.0%)	0.99 (0.82 - 1.20; 0.949)
Substance-induced psychiatric disorders [e]	36 / 150 (24.0%)	0.63 (0.41 - 0.97; 0.038*)
Mood disorders [f]	45 / 143 (31.5%)	0.65 (0.44 - 0.98; 0.038*)
Other psychiatric disorders	281 / 714 (39.4%)	Ref.
Mood disorders [f]	45 / 143 (31.5%)	0.63 (0.41 - 0.95; 0.028*)
Other psychiatric disorders	253 / 697 (36.3%)	Ref.
Anxiety and other nonpsychotic disorders [g]	73 / 160 (45.6%)	1.65 (1.11 - 2.44; 0.013*)
Other psychiatric disorders	317 / 834 (38.0%)	Ref.
Intellectual disabilities [h]	9 / 23 (39.1%)	3.21 (1.24 - 8.29; 0.016*)

[a] Adjusted for age, sex, hospital, smoking, obesity, number of medical conditions, as well as hydroxychloroquine, azithromycin, tocilizumab, and dexamethasone.
[b] Assessed using ICD-10 diagnosis codes for mental and behavioral disorders due to psychoactive substance use (F10-F19).
[c] Assessed using ICD-10 diagnosis codes for mood [affective] disorders (F30-F39).
[d] Assessed using ICD-10 diagnosis codes for anxiety, dissociative, stress-related, somatoform and other nonpsychotic psychiatric disorders (F40-F49).
[e] Assessed using ICD-10 diagnosis codes for intellectual disabilities (F70-F79).
* Two-sided p-value is significant (p<0.05).
Abbreviations: AOR, adjusted odds ratio; CI, confidence interval.

Supplemental References

1. Jouffroy J, Feldman SF, Lerner I, Rance B, Neuraz A, Burgun A (2020): MedExt: combining expert knowledge and deep learning for medication extraction from French clinical texts.

2. Williamson EJ, Walker AJ, Bhaskaran K, Bacon S, Bates C, Morton CE, *et al.* (2020): Factors associated with COVID-19-related death using OpenSAFELY. *Nature*, vol. 584. pp 430–436.
